# A haplotype-resolved genome assembly of *Cardamine amara*

**DOI:** 10.1038/s41597-026-08540-1

**Published:** 2026-11-11

**Authors:** Rhianah Sandean, Marek Šlenker, Denzel Renner, Gabriela Šrámková, Judita Zozomová-Lihová, Karol Marhold, Terezie Mandáková, Levi Yant

**Affiliations:** 1 Linnean Centre for Plant Biology, Department of Plant Biology, Uppsala BioCenter, Swedish University of Agricultural Sciences, Uppsala, Sweden; 2 Department of Botany, Faculty of Science, Charles University, Prague, Czech Republic; 3 Plant Science and Biodiversity Centre, Slovak Academy of Sciences, Bratislava, Slovakia; 4 Department of Experimental Biology, Faculty of Science, Masaryk University, Brno, Czech Republic; 5 Central European Institute of Technology, Masaryk University, Brno, Czech Republic

## Abstract

The genus *Cardamine* (Brassicaceae) includes several well-established model species widely used in evolutionary biology, ecology, and developmental studies. While a chromosome-scale reference is available for *Cardamine hirsuta*, its close relative *Cardamine amara*, with unique ecological and reproductive traits, as well as a tendency toward polyploidy, has lacked genomic resources. This gap has limited genomic investigation of an otherwise extensively studied system. Here, we present two chromosome-scale, haplotype-resolved genome assemblies for a *C. amara* individual from Velký Rečkov, Czech Republic, generated using 444-fold PacBio HiFi long-read data and 320-fold Hi-C data. The two haplomes span 211.96 Mb and 212.03 Mb, with scaffold N50s of 27.01 Mb and 26.41 Mb, respectively. Gene space completeness is high in both assemblies (complete BUSCOs ≥ 99.6%). Comparative analyses confirmed a major pericentromeric inversion between *C. amara* and *C. hirsuta* and revealed large structural variants even between haplomes within this single heterozygous individual. These high-quality assemblies provide essential genomic references that will enable deeper insights into genome structure, evolution, and adaptation across the diverse *Cardamine* genus.

## Background & Summary

**Fig. 1:**
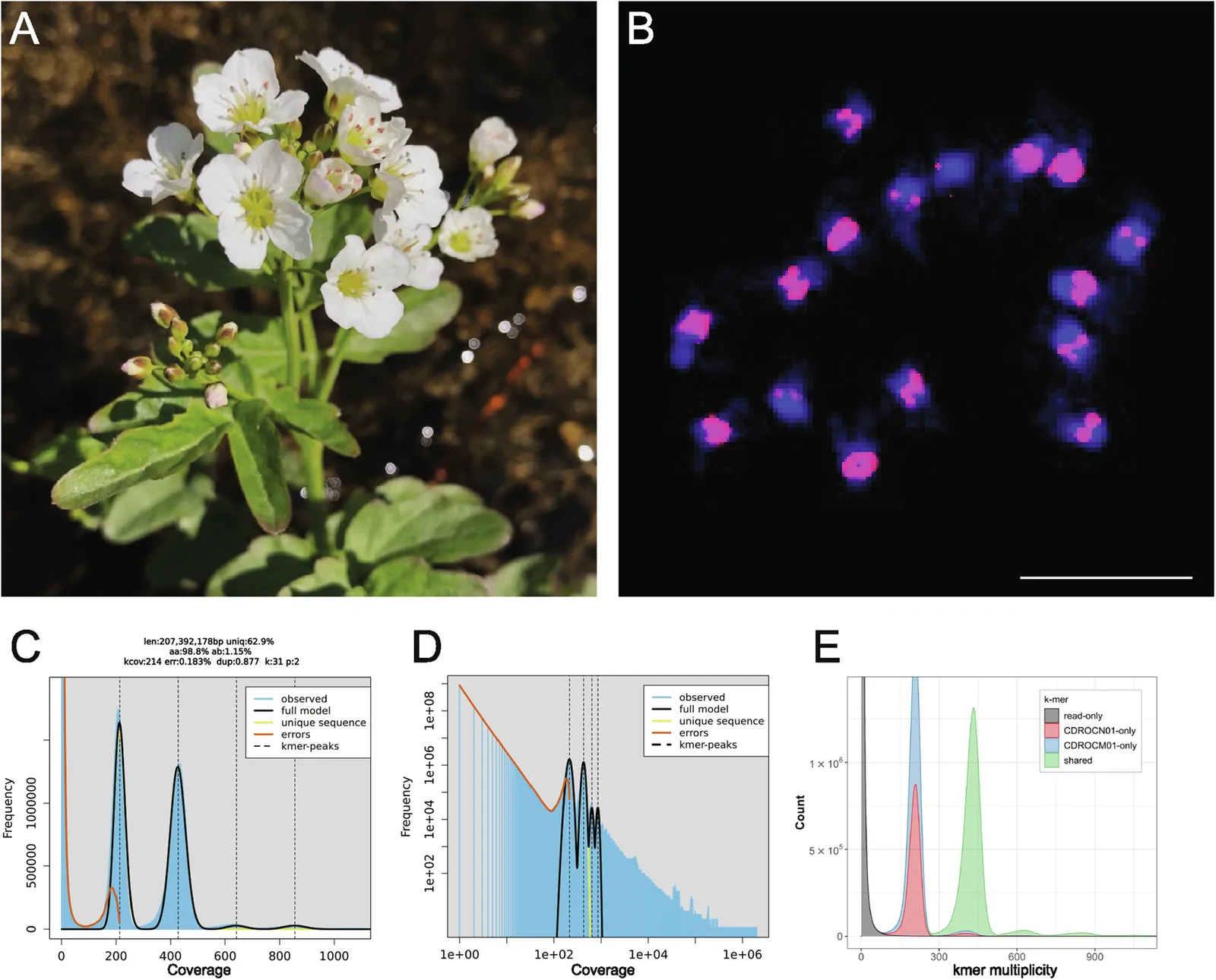
*Cardamine amara*, physical and genomic features. (**A**) Flowering *C. amara* plant. (**B**) Mitotic chromosomes of *C. amara*, isolated from root tips and hybridized with a probe specific to the *C. amara*-exclusive 290 bp centromeric tandem repeat (red fluorescence). Chromosomes were counterstained with DAPI. Scale bar: 10 μm. (**C**,**D**) k-mer profiles using Genomescope 2.0 (31-mers based on HiFi reads). Linear X-axis (**C**) emphasises high heterozygosity, while Log X-axis (**D**) reveal high coverage repeat peaks. (**E**) shows the k-mer distribution plot in Merqury.

The genus *Cardamine* (Brassicaceae) comprises almost 300 species^1^ and serves as a valuable system for investigating plant evolution, ecology, and developmental biology. Among its members, the self-compatible annual weed *Cardamine hirsuta* has become a leading model for studying morphological diversification and regulatory evolution (e.g.^2–4^), supported by the availability of a chromosome-scale genome^2^. In contrast, its close relative *Cardamine amara* (Fig. 1A) has remained underrepresented in genomic resources, despite its status as a progenitor in both auto- and allopolyploid lineages and its use in comparative evolutionary studies of whole-genome duplication^5,6^ and ecological adaptation. Unlike *C. hirsuta*, *C. amara* is a mesic-adapted and submergence-tolerant perennial^7^ with a combination of sexual reproduction with androdioecy (coexistence of hermaphrodite and female-sterile individuals) and vegetative reproduction by creeping stolons^8^. *C. amara* is a widespread Eurasian species that is predominantly diploid (2n = 2x = 16, Fig. 1B) but includes autopolyploids in the Alps and Sudetes^9^ and has also been identified as the progenitor of several allopolyploids and hybrids across Europe^10–13^. Thus *C. amara* presents a powerful genomic model for studying the genetic basis of various traits relevant to plant ecology, reproductive biology, polyploid evolution and speciation.

To provide a quality genomic reference in *C. amara*, we here generate chromosome-scale genome assemblies of both haplomes from a single highly heterozygous *C. amara* individual. These assemblies integrate 444-fold PacBio HiFi sequencing with 320-fold Illumina-based Hi-C data. Each haplome assembly spans 212 Mb and consists of eight pseudomolecules, with scaffold N50 values of 27.0 Mb and 26.4 Mb, respectively. BUSCO^14^ analysis revealed 99.6% completeness in haplome 1 (S: 97.1%, D: 2.4%) and 99.7% in haplome 2 (S: 97.6%, D: 2.1%), indicating excellent gene space representation. These assemblies also capture extensive interhaplome structural variation, including large inversions and complex repetitive regions such as a telomeric NOR exceeding 8 Mb.

These resources provide a genomic foundation for *Cardamine* and Brassicaceae researchers, enabling studies of genome architecture, structural heterozygosity, and adaptation. By facilitating comparative and functional analyses across diploid and polyploid taxa, these genomes contribute to broader efforts in plant evolutionary genomics and systematics. With its growing role in studies spanning ecological genomics, chromosomal evolution, reproductive and developmental biology, *Cardamine* is increasingly positioned as a multipurpose model for plant research.

## Methods

### Plant material, nucleic acid extraction and sequencing

High molecular weight (HMW) DNA was extracted from a young seedling of *C. amara* accession Velký Rečkov, CZ (50.4983222 N, 14.9031061 E) using a modified CTAB DNA extraction protocol^15^. A herbarium voucher was prepared from the sequenced plant and deposited in the Herbarium of the Department of Botany, Charles University, Prague under the accession number PRC 488467. HMW DNA was confirmed by gel electrophoresis and sheared to the appropriate size range (15–20 kb) and PacBio HiFi sequencing libraries were constructed by Novogene (UK) Limited. Sequencing was performed on 1 full SMRT cell of the PacBio Sequel Revio system in CCS mode to generate 94.2 Gb (~444-fold coverage) of HiFi reads (read n50 = 15.4 Kb). From the same plant one Hi-C library was prepared using leaf samples, using the Arima Genomics® Hi-C kit (Carlsbad, California, USA) according to the manufacturer’s protocol. The Hi-C library was sequenced on an Illumina NovaSeq 6000 S4 platform to generate paired end 150 bp reads (67.9 Gb raw data; ~320-fold coverage). Plants were grown in a glasshouse in pots containing natural soil and maintained at natural conditions. All sequencing was carried out by Novogene (UK) Limited.

### Chromosome preparation and genome size estimation

Chromosome preparation was performed from root tips as described^16^. The size of the *C. amara* genome was estimated by using k-mer (k = 31) distribution generated with KMC^17^ on the HiFi reads and by flow cytometry^18^. A k-mer count histogram was generated using GenomeScope (v2.0)^19^ with parameters: ploidy = 2, k-mer length = 31, max k-mer coverage = 200000, to account for the high coverage repeats discovered during genome size estimation. The *C. amara* genome was estimated as ~208 Mb with heterozygosity of 1.15% (Fig. 1C and D), highly congruent with the flow cytometric 2C estimate of 0.42 pg. Haploid k-mer coverage estimate was 214, with the diploid peak at 428, consistent with the estimated target sequencing depth of ~444-fold (Fig.1C and D).

### Chromosome-scale assemblies of two phased haplomes

We first assembled HiFi reads into the initial set of contigs using hifiasm (v0.24)^20^ with default parameters for integrated Hi-C, and we assessed assembly metrics using gfastats (v1.3.1)^21^. We identified likely contaminant contigs (those not categorised as Streptophyta or ‘no-hit’) using NCBI-fcs^22^ and Blobtools^23^ and removed them if their remapped HiFi read coverage was >2 × or <0.5 × the genome-wide mean.

**Fig. 2:**
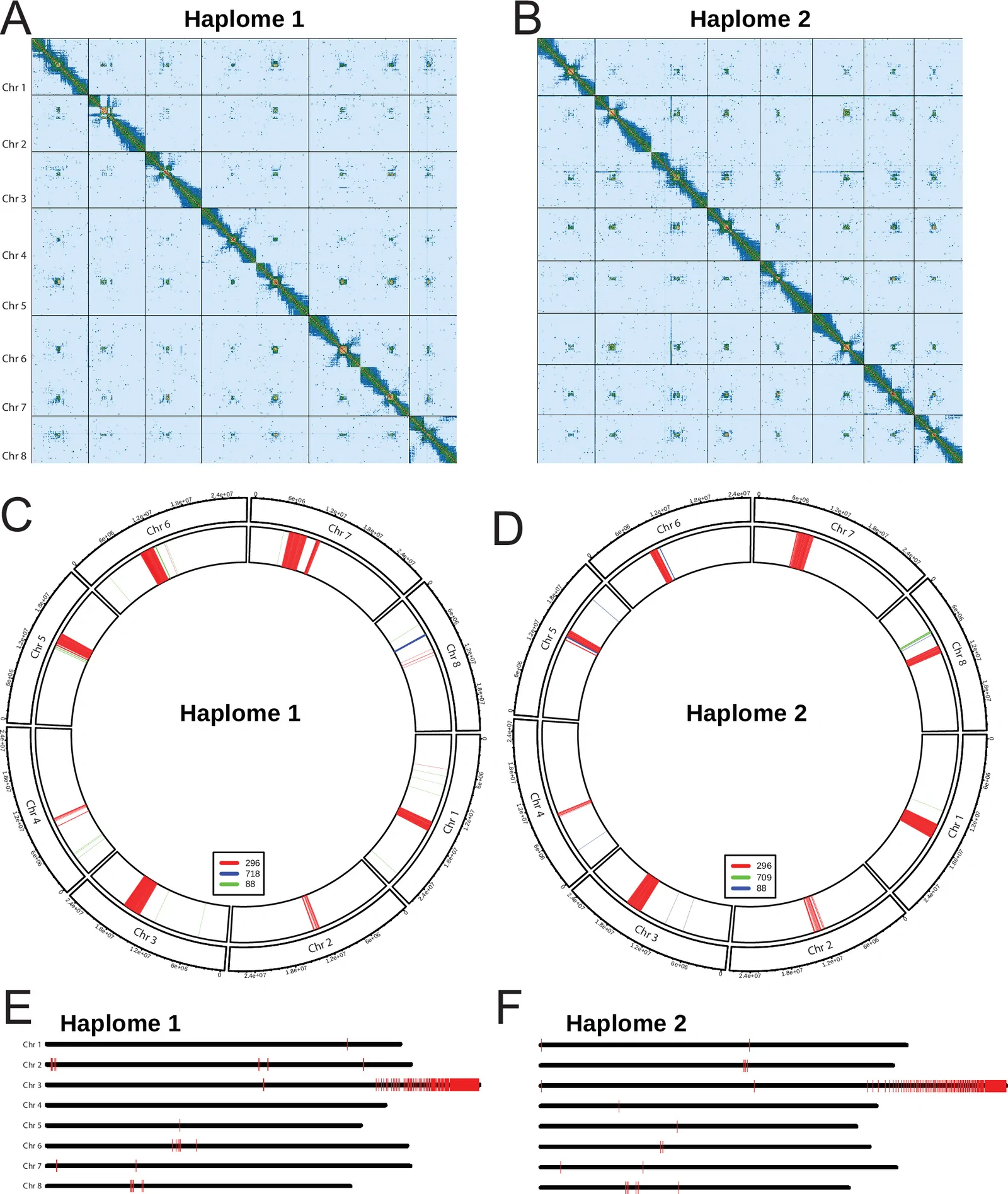
*Cardamine amara*, dual haplome assemblies, centromere repeats, and interhaplome conservation. (**A**,**B**) Hi-C contact maps illustrating contiguous chromosome builds and centromeric reflectivity in the trans space. (**C**,**D**) Centromere-diagnostic repeats are well conserved between haplomes, as represented by analysis in Tandem Repeat Annotation and Structural Hierarchy (TRASH) analysis. (**E**,**F**) gaps are shown red lines, with the vast majority in the ultra-repetitive NOR regions of chromosome 3.

To achieve a chromosome-level assembly, we then scaffolded each haplome by mapping the Hi-C data onto the contig assembly using BWA-MEM (v0.7.18)^24^ and the resulting alignments were processed following the Dovetail Omni-C pipeline^25^. The filtered bam file was used as input for YaHs (v1.2.2)^26^ with default parameters to scaffold the assembly. To annotate features on the contact map, telomere tracks were generated using tidk (v0.2.1)^27^ and gap tracks were created using Bedtools (v2.31.1)^28^ genomecov. These were visualised by PretextGraph (https://github.com/sanger-tol/PretextGraphv0.0.6)^29^ and the chromatin contact matrix was manually corrected using PretextView (v0.2.5) by following the Rapid Curation pipeline (Fig. 2A and B; https://gitlab.com/wtsi-grit/rapid-curation).

We then assessed both curated assemblies using gfastats as above and the compleasm (v0.2.5)^30^ BUSCO implementation (Table 1). Each final assembly consists of 8 pseudomolecules, with scaffold N50s of 27.0 Mb and 26.4 Mb, corresponding to the size of each full chromosome. Despite the very high heterozygosity indicated above in the k-mer analysis, both haplome sizes were very similar to one another (211.96 Mb and 212.03) and within 2% of the k-mer-based inferred genome size estimate of 208 Mb. Overall metrics of both haplomes were highly concordant with one another (Tables 1 and 2). The orientation and the chromosome name of each pseudomolecule were determined based on synteny with one another using dotplot comparison of sequence alignments produced by MUMmer’s (v3.23)^31^ nucmer aligner and viewed on Dot^32^ as well as SyRI (v1.7.1)^33^ and GENESPACE (v1.2.3)^34^. Centromere and telomere locations were confirmed in Pretext (Fig. 2A and B) and TRASH (v0.1)^35^ (Fig. 2C and D). Overall, both assemblies were well ordered and contained few gaps, with the exception of a large nucleolus organiser region (NOR) on chromosome 3 (Fig. 2E and F; Table 2).

**Table 1:** Summary metrics for both *Cardamine amara* assemblies.

	Haplome 1 (including NORs)	Haplome 2 (including NORs)
Number of scaffolds	8	8
Total length (bp)	211,965,902	212,030,739
Scaffold N50 (bp)	27,013,139	26,410,944
Largest scaffold (bp)	32,406,040	34,926,063
Contig N50 (bp)	15,519,148	13,424,908
Largest contig (bp)	25,339,093	19,278,808
GC content (%)	36.3	36.5
BUSCOs (%)	Complete: 99.6% Single copy: 97.1% Duplicate: 2.4% Missing: 0.4% *n = *4,596	Complete: 99.7% Single copy: 97.6% Duplicate: 2.1% Missing: 0.3% *n = *4,596

**Table 2:** Lengths of each chromosome in both *Cardamine amara* assemblies.

Chromosome	Haplome 1 length (bp)	Haplome 2 length (bp)	Haplome 1 gaps	Haplome 2 gaps	Haplome 1 gaps (bp)	Haplome 2 gaps (bp)
1	26,464,913	27,436,750	1	2	100	300
2	27,271,854	26,410,944	11	4	2,200	800
3	32,406,040	34,926,063	2 + 144 (NOR)	2 + 139 (NOR)	29,200	28,000
4	25,339,093	25,168,896	0	1	0	100
5	23,511,462	23,663,108	1	1	200	200
6	27,013,139	24,651,808	6	2	1,000	400
7	27,238,142	26,660,122	3	2	600	300
8	22,721,259	23,113,048	7	6	1,300	1,100

### Structural variation between haplomes and vs. *Cardamine hirsuta*

**Fig. 3:**
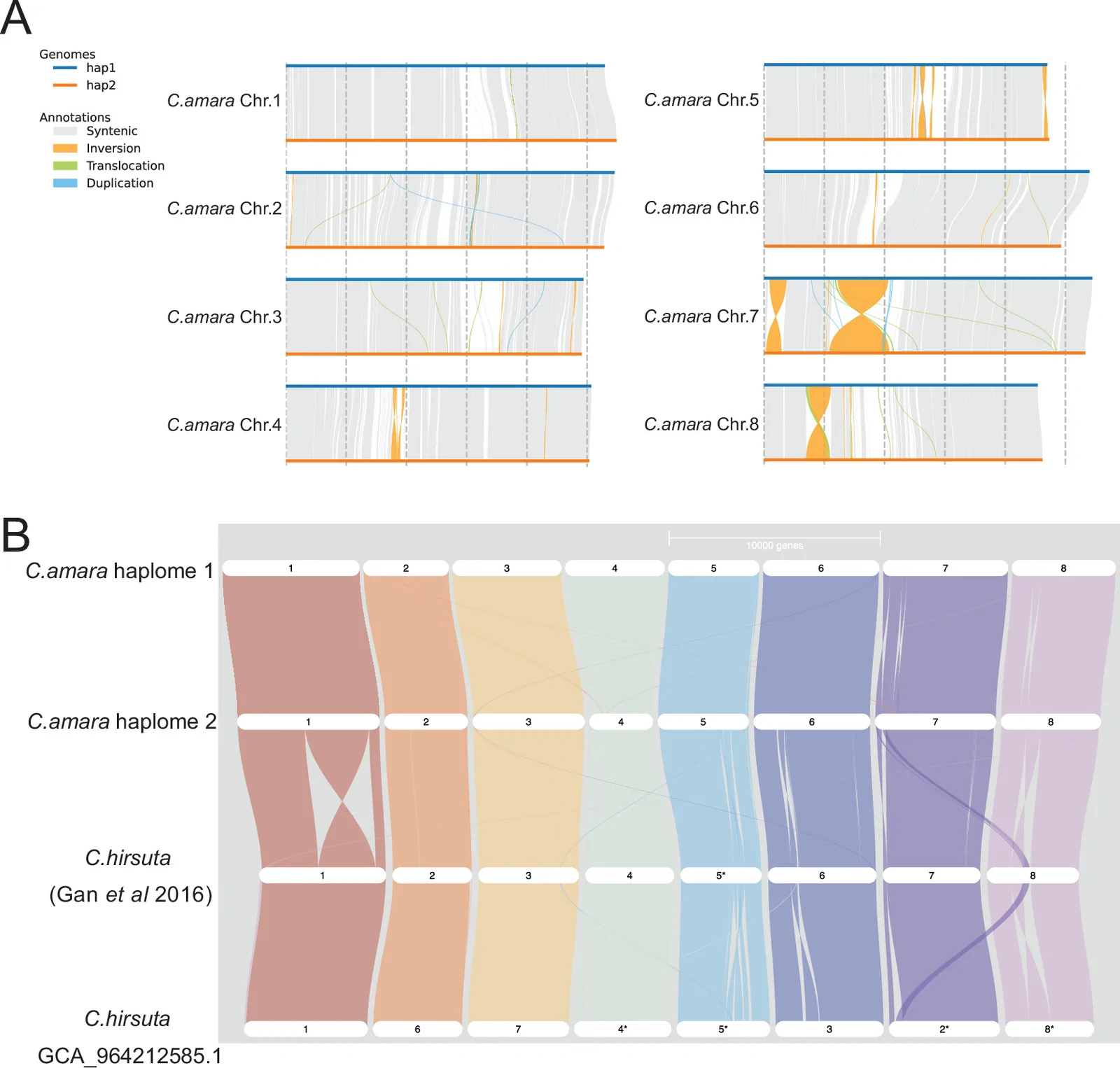
*Cardamine amara* interhaplome conservation and divergence vs. *Cardamine hirsuta*. (**A**) Coordinate-based synteny illustration of interhaplome structural variation, with major inversions illustrated in orange. (**B**) Gene-content-scaled structural variation recapitulates most inversions between the two *C. amara* haplomes and additionally shows major structural variation between *C. amara* and *C. hirsuta*. NORs are removed from (A).

Synteny and collinearity analysis of the two haplomes were performed with several methods. Nucmer-based dotplots indicated major inversions between the haplomes in chromosomes 7 and 8, with several smaller inversions also in chromosomes 2, 3, 4, 5, and 6. These were confirmed in comparative analysis in SyRI (Fig. 3A). As expected, only a subset was confirmed in GENESPACE-based comparisons (Fig. 3B), which also contrasted in scaling, as the latter analyses are based on annotated gene synteny, rather than comparisons of genomic coordinates in the Nucmer and SyRI-based comparisons. Overall, there was broad synteny between the two haplomes, and we observed no major translocations between them. Centromere repeat locations were very consistent between the two haplomes (Fig. 2C and D), as was also reflected in the HiC contact maps (Fig. 2A and B). We additionally show this 290-bp centromeric tandem repeat cytologically in Fig. 1B.

Comparing both of our *C. amara* haplome builds to the standard reference *C. hirsuta* assembly^2^, we observed a major inversion in *C. amara* chromosome 1 and *C. hirsuta* rl_1 (Fig. 3B), corresponding to a known pericentric inversion^11^. Aside from this, both of our haplomes harbored several major structural variations relative to the *C. hirsuta* assembly. Consulting a second *C. hirsuta* assembly (Fig. 3B), we confirmed that these variations are indeed species-specific and not artefactual to any one reference.

### Annotation and genomic distribution of rDNA

**Fig. 4:**
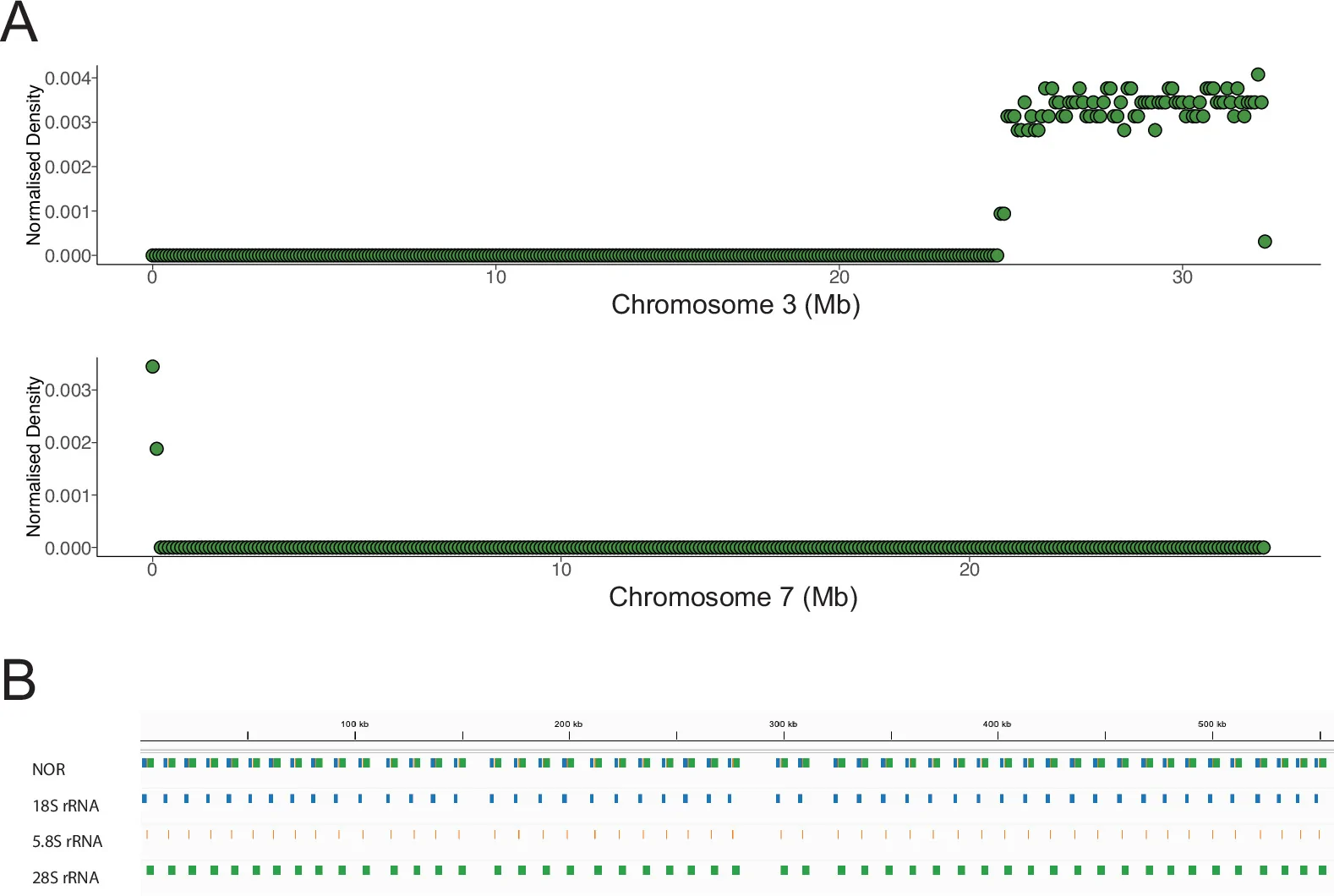
rDNA gene density is strongly concentrated on Chromosome 3 NORs in large repeat arrays. (**A)**. Relative density of rRNA-encoding rDNA genes is concentrated on the south arm of Chromosome 3. Only Chromosomes 3 and 7 of one haplome are shown, as normalised signal is absent in the rest of the example haplome. We computed the normalised rDNA gene density across non-overlapping 100 kb windows for all chromosomes for each haplome, where green dots indicate relative density of all rRNA encoding loci (18S, 5.8S, and 28S). (**B**) Example rDNA gene repeat array in haplome 2 NOR. Shown is the single largest contig in the NOR of Haplome 2.

To identify ribosomal RNA (rRNA)-encoding genes in both *C. amara* haplomes, we used barrnap (v0.9)^36^, specifying the –kingdom argument to “eukaryote” and applying a default e-value threshold of ≤1e-6. These predictions, combined with inspection of Hi-C contact maps, allowed identification of an ~8 Mb ribosomal DNA (rDNA) terminal position of chromosome 3 in both haplomes (an example is presented in Fig. 4A). We also identified an additional <200 kb rDNA repeat array at the telomeric end of chromosome 7 in both haplomes (Fig. 4A). The major NOR region on chromosome 3 comprises approximately equal copy numbers of 18S, 5.8S, and 28S rDNA sequences (Table 3 values are haplome-wide, but nearly all 18S, 5.8S and 28S copies lie in this NOR), although the number of distinct sequence variants (rDNA gene subtypes) increased with rDNA gene length. For each rDNA gene type, copy number was largely dominated (sometimes overwhelmingly) by a single sequence variant. This overrepresentation was confirmed with k-mer-based approaches. Thus, this region appears to be a very large nucleolus organiser region (NOR), similar to those described in some grasses and, only recently, in *Arabidopsis thaliana*^37^.

**Table 3:** Summary metrics for rRNA-encoding loci in both *Cardamine amara* assemblies.

Haplome	Locus	Copy number	Unique variants	Average length (bp)	Total length (bp)	% of Haplome
1	5.8S	799	23	154	122,708	0.06%
	18S	829	175	1,758	1,457,103	0.69%
	28S	833	362	3,550	2,957,065	1.40%
	5S	730	167	105	76,968	0.04%
2	5.8S	1,044	22	154	160,593	0.08%
	18S	1,075	215	1,762	1,894,470	0.89%
	28S	1,086	442	3,567	3,873,796	1.83%
	5S	852	195	105	89,274	0.04%

In contrast to the overall well-ordered and low-gap assembly, the NOR region was fragmented, consisting of 144 contigs in haplome 1 and 139 contigs in haplome 2. However, many contigs were large and intact, giving information about rDNA array structure. For each haplome, we extracted the largest contig within the NOR and visualised array structure, revealing a tandemly repeated arrangement of 18S-5.8S-28S genes in the same orientation (Fig. 4B).

### Repeat identification

Repetitive sequences were identified using a combination of *de novo* and homology-based approaches. For the *de novo* prediction, we constructed a custom repeat library employing HelitronScanner (v1.1)^38^, LTR_FINDER (v1.3)^39^, LTR_retriever (v3.0.1)^40^, MiteFinderII (v1.0.006)^41^, RepeatModeler (v2.0.6)^42^, and SINE_Scan (v1.01)^43^. The resulting repeat elements were classified using RepeatClassifier (v2.0.6)^42^ and subsequently merged with known repeat elements from the Dfam (v3.9)^44^ and RepBase (v20181026)^45^ databases, queried for the Brassicaceae-associated repeat elements. Next, vsearch (v2.30.0)^46^ was used to cluster repeat elements and to generate a non-redundant repeat library. Finally, RepeatMasker (v.4.1.8)^42^ was used to mask the genome with the finalized repeat library. By this approach, we identified a total of 143,818,698 bp and 144,298,706 bp repetitive sequences for haplome 1 and haplome 2 assemblies, respectively, accounting for approximately 67.85% and 68.06% of each haplome. Detailed statistics are provided in Table 4.

**Table 4:** Summary of *Cardamine amara* repeat annotations.

Annotation Type	Haplome 1			Haplome 2		
	Number	Length (bp)	Percent (%)	Number	Length (bp)	Percent (%)
LTR	88,188	80,634,566	38.04	87,824	87,322,213	41.18
SINEs	1,871	415,138	0.20	1,923	445,947	0.21
Penelope	39	4,681	0.00	50	7,544	0.00
LINEs	7,533	3,325,229	1.57	7,497	3,377,682	1.59
DNA transposons	45,143	14,858,136	7.01	44,543	14,448,958	6.81
Rolling-circles	7,530	2,469,307	1.16	7,313	2,293,719	1.08
Unclassified	117,195	35,478,581	16.74	119,792	35,825,522	16.90
Total		143,818,698	67.85		144,298,706	68.06

### Evidence guided gene prediction

Prediction of protein-coding genes was performed using the repeat-masked genome with the combination of *ab initio*, homology-based, and transcriptome-based predictions. The *ab initio* prediction was carried out using BRAKER3 (v3.0.8)^47^. BRAKER automatically trained Augustus (v3.5.0)^48^ and GeneMark-ETP (v1.02)^49^ based on RNA sequence alignments and reference proteins obtained from OrthoDB12’s Viridiplantae dataset^14^. The RNA-seq data of *C. amara* (NCBI accession numbers: SRR11977918, DRR111001, DRR166629-DRR166631, DRR216635-DRR216643, SRR26392757) were processed to remove low-quality reads using fastp (v0.20.1)^50^ and aligned to the genome using HISAT2 (v2.2.1)^51^. For the homology-based approach, GeMoMa (v1.9)^52^ was used to generate gene models based on protein sequences from seven related species: *Cardamine hirsuta*^2^*, Arabidopsis arenosa* (GCA_905216605.1), *Arabidopsis thaliana* (GCF_000001735.4), *Brassica napus* (GCF_020379485.1), *Brassica rapa* (GCF_000309985.2), *Raphanus sativus* (GCF_000801105.2), and *Thlaspi arvense* (GCA_911865555.2). For the transcriptome-based approach, the transcriptome was assembled using StringTie (v2.2.1)^53^, and coding regions were predicted using TD2 (v1.0.5)^43^. Gene models from all three approaches were integrated using EvidenceModeler (v2.1.0)^54^ to produce the high-confidence set of protein-coding gene annotations. Finally, PASA (v2.5.3)^55^ was used to update gene structures with untranslated regions and alternative splicing variation information. Combining the above steps, 25,572 and 25,330 protein-coding genes were finally annotated in haplome 1 and haplome 2, respectively (Table 5).

**Table 5:** Summary of protein-coding genes in both *Cardamine amara* assemblies.

	Haplome 1	Haplome 2
Number of protein-coding genes	25,572	25,330
Mean gene length (bp)	2,454	2,405
Number of predicted protein sequences	40,773	38,962
Mean protein length (aa)	419	421
Number of exons per gene	6.2	6.2
Mono:multi -exonic ratio	0.14	0.15
Mean exon length (bp)	305	300
Number of introns per gene	5.2	5.2
Mean intron length (bp)	182	182
Number of CDSs per gene	1.6	1.5
Mean CDSs length (bp)	1,257	1,262

### Functional annotation

Functional annotation of the protein-coding genes was conducted by aligning protein sequences against multiple databases, including Kofam^56^, RefSeq^27^, SwissProt, and TrEMBL^57^, using diamond (v2.1.11.165)^58^, with e-value ≤1e-5 as threshold. Furthermore, eggNOG-mapper (v2)^59^ was utilized to search for homologous genes in the eggNOG database, to add KEGG terms, Gene Ontology terms, and Pfam domains. According to this approach, a total of 99.62% (25,476 out of 25,572) and 99.71% (25,256 out of 25,330) of protein-coding genes were successfully annotated in the aforementioned databases for haplome 1 and haplome 2, respectively (Table 6).

**Table 6:** Summary of the *Cardamine amara* genome gene functional annotation.

Database	Haplome 1		Haplome 2	
	Number	Percent (%)	Number	Percent (%)
EggNOG	24,615	96.26%	24,432	96.45%
Kofam	18,538	72.49%	18,417	72.71%
RefSeq	25,425	99.43%	25,194	99.46%
Swiss-Prot	19,882	77.75%	19,806	78.19%
TrEMBL	25,460	99.56%	25,238	99.64%
Total	25,476	99.62%	25,256	99.71%

## Data Records

The raw PacBio HiFi and Hi-C sequence reads have been deposited in the NCBI Sequence Read Archive (SRA) under BioProject accession PRJNA1415849 and study accession numbers SRP727368^60^ and SRP727327^61^. The chromosome-scale genome assemblies and annotations are available in GenBank under assembly accession numbers GCA_056512385.1^62^ and GCA_056512305.1^63^.

## Technical Validation

**Fig. 5:**
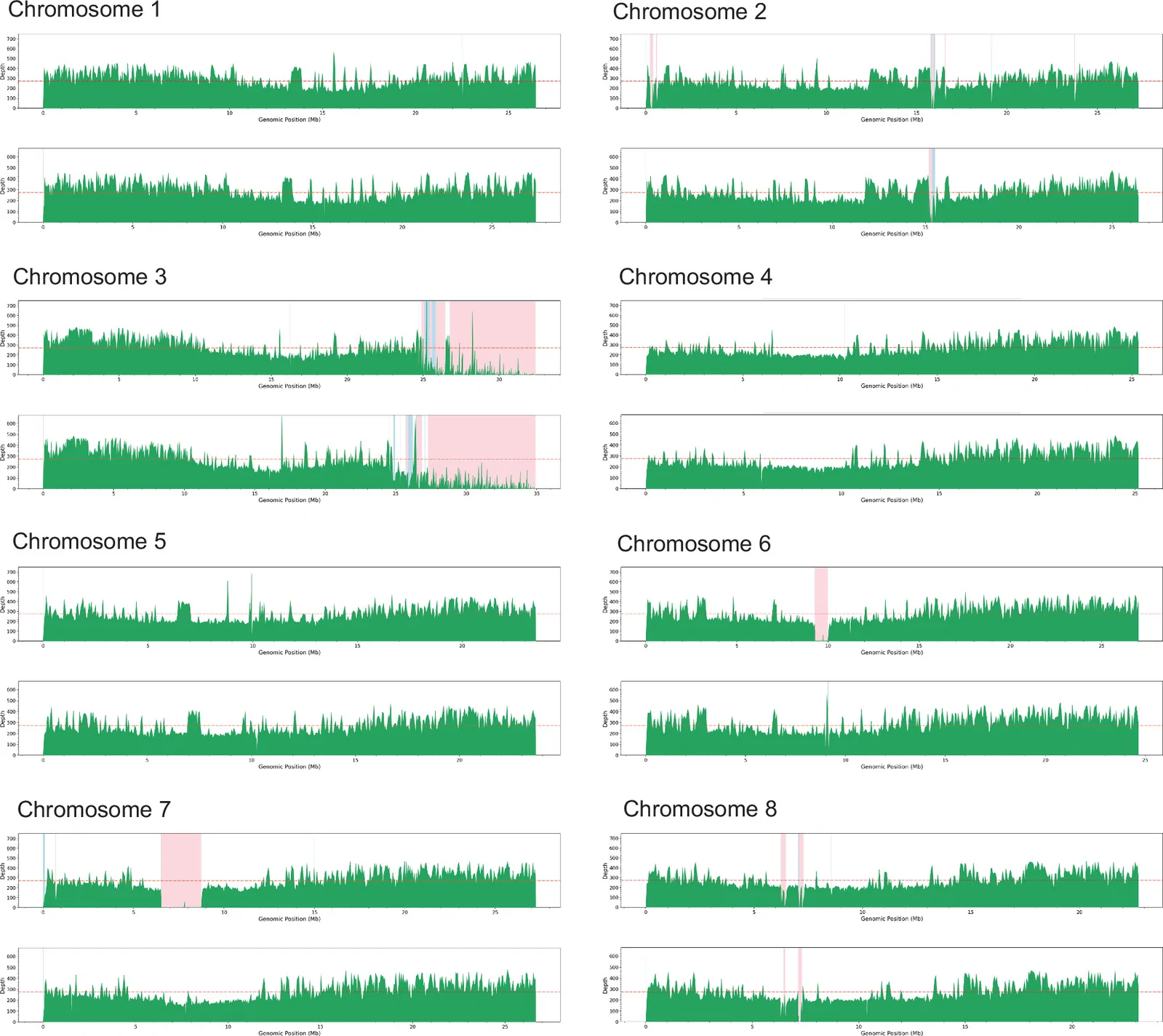
HiFi read-mapping depth across the two *C*. *amara* haplomes. HiFi reads were mapped independently to each haplome assembly and read depth per 50 kb windows was calculated across each chromosome. Each panel shows the corresponding chromosome in haplome 1 (upper track) and haplome 2 (lower track). Shaded regions indicate regions of atypical read depth that overlap regions of high repeat content, typically associated with centromeric regions.

Four primary criteria were used to evaluate the correctness and completeness of the *C. amara* genome assemblies. First, the final curated assemblies were assessed by mapping the Hi-C reads to the post-curated assemblies and generating a final Hi-C contact map using PretextMap, which were visualised using PretextViewAI (v1.0.3)^29^. This showed very clear, dense cis patterns along the diagonal of both haplomes, indicating no potential misassemblies (Fig. 2A and B). Second, compleasm^30^ implementation of BUSCOs (-l brassicales_odb10) gave very high percentages of complete BUSCOs: haplome 1 contained 99.55% complete BUSCOs (S:97.13%; D:2.42%; M:0.37%), while haplome 2 contained 99.65% complete BUSCOs (S:97.56%; D:2.09%; M:0.28%; n:4,596). These results indicate a very high completeness of both *C. amara* haplomes. Third, the quality of both haplomes was assessed with Merqury (v1.3)^64^ based on the PacBio HiFi reads using 31-mers. For the haplomes together, the QV (consensus quality value) score was 65.6 and the k-mer completeness was 99.3%, indicating an extremely high base quality and overall completeness. Fourth, to evaluate assembly uniformity and identify potential collapsed or over-duplicated regions, HiFi read-mapping depth was also assessed across all chromosomes in both haplomes. Winnowmap2 (v2.03)^65^ and Minimap2 (v2.30)^66^ were used to align the HiFi reads to the genome assembly and generate coverage plots using GCI (Genome Continuity Inspector) (v0.14.0)^67^ with the mapq60 parameter. GCI plots demonstrated high concordance between haplomes, and manual inspection of regions with atypical depth overlapped with regions enriched with simple repeats (Fig. 5).

To evaluate the completeness of the genome annotation, BUSCO (v5.8.2)^14^ and OMArk (v0.3.0)^68^ analyses were performed on the annotated gene set using the brassicales_odb10 and LUCA.h5 databases. BUSCO analysis identified 98.1% and 98.3% complete conserved genes in annotations of haplomes 1 and 2, respectively, with an overall completeness of 99.0% (Table 7). Consistently, OMArk reported annotation completeness of 94.74%, 94.81%, and 96.16% for haplome 1, haplome 2, and the combined genome, respectively. No contaminant sequences were detected, and only a small proportion of proteins remained unclassified, further supporting the high quality and reliability of the annotation (Table 7). In addition, PSAURON (v1.1.3)^69^ yielded scores of 97.76% for both haplomes. Furthermore, featureCounts (v2.1.1)^70^ showed that 95.07% and 94.83% of multiexonic genes in haplomes 1 and 2, respectively, were supported by splice-junction evidence from mapped RNA-seq reads, with an average coverage of at least one read per exon. The number of gene models (~25k) is comparable with the number of annotated protein-coding genes in *C. hirsuta* (~29,500) and *A. thaliana* (~27,500).

**Table 7:** Quality control of predicted genes in the *Cardamine amara* genome annotations.

Program	Features	Haplome 1		Haplome 2		Genome	
BUSCO	Complete BUSCOs	4,511	98.1%	4,520	98.3%	4,552	99.0%
	Complete and single-copy BUSCOs	4,351	94.6%	4,370	95.0%	—	—
	Complete and duplicated BUSCOs	160	3.5%	150	3.3%	—	—
	Fragmented BUSCOs	13	0.3%	9	0.2%	—	—
	Missing BUSCOs	72	1.6%	67	1.5%	38	0.8%
	Total BUSCO groups searched	4,596					
OMArk	Completeness	94.74%		94,81%		96,16%	
	Consistency	96.06%		96.13%		96.09%	
	Contaminants	0.00%		0.00%		0.00%	
	Unknown	2.35%		2.44%		2.39%	
featureCounts		95.07%		94.83%		—	

## Data Availability

Custom code generated for these analyses is available at https://github.com/denzelrenner/Cardamine-Annotation, https://github.com/rsandean/C.amara_genome_assembly.git, and https://github.com/MarekSlenker/Code-Availability/tree/main/Sandean_et_al_2026_Sci_Data.
